# Alleviating the effect of quinoa and the underlying mechanism on hepatic steatosis in high-fat diet-fed rats

**DOI:** 10.1186/s12986-021-00631-7

**Published:** 2021-12-18

**Authors:** Chenwei Song, Wei Lv, Yahui Li, Pan Nie, Jun Lu, Yanlou Geng, Zhang Heng, Lihua Song

**Affiliations:** 1 School of Agriculture and Biology, Shanghai Jiao Tong University, Shanghai, 200240 China; 2 National Semi-Arid Agriculture Engineering Technology Research Center, Shijiazhuang, 050051 Hebei China; 3 Center for Food Evaluation, State Administration for Market Regulation, Beijing, 100070 China; 4 Shanghai Center for Plant Stress Biology, CAS Center for Excellence in Molecular Plant Sciences, Chinese Academy of Sciences, Shanghai, 201602 China

**Keywords:** Quinoa, Fatty liver, Oxidative stress, Lipid metabolism, Immune response

## Abstract

**Background:**

Nonalcoholic fatty liver disease (NAFLD) is considered the hepatic component of metabolic syndrome and has attracted widespread attention due to its increased prevalence. Daily dietary management is an effective strategy for the prevention of NAFLD. Quinoa, a nutritious pseudocereal, is abundant in antioxidative bioactive phytochemicals. In the present study, the effects of different amounts of quinoa on the progression of NAFLD and the related molecular mechanism were investigated.

**Methods:**

Male SD rats were simultaneously administered a high fat diet (HF) and different amounts of quinoa (equivalent to 100 g/day and 300 g/day of human intake, respectively). After 12 weeks of the intervention, hepatic TG (triglyceride) and TC (total cholesterol) as well as serum antioxidative parameters were determined, and hematoxylin–eosin staining (H&E) staining was used to evaluate hepatic steatosis. Differential metabolites in serum and hepatic tissue were identified using UPLC-QTOF-MS^E^. The mRNA expression profile was investigated using RNA-Seq and further verified using real-time polymerase chain reaction (RT-PCR).

**Results:**

Low amounts of quinoa (equivalent to 100 g/d of human intake) effectively controlled the weight of rats fed a high-fat diet. In addition, quinoa effectively inhibited the increase in hepatic TG and TC levels, mitigated pathological injury, promoted the increase in SOD and GSH-Px activities, and decreased MDA levels. Nontarget metabolic profile analysis showed that quinoa regulated lipid metabolites in the circulation system and liver such as LysoPC and PC. RNA-Seq and RT-PCR verification revealed that a high amount of quinoa more effectively upregulated genes related to lipid metabolism [Apoa (apolipoprotein)5, Apoa4, Apoc2] and downregulated genes related to the immune response [lrf (interferon regulatory factor)5, Tlr6 (Toll-like receptor), Tlr10, Tlr11, Tlr12].

**Conclusion:**

Quinoa effectively prevented NAFLD by controlling body weight, mitigating oxidative stress, and regulating the lipid metabolic profile and the expression of genes related to lipid metabolism and the immune response.

**Supplementary Information:**

The online version contains supplementary material available at 10.1186/s12986-021-00631-7.

## Introduction

Currently, a high-fat (HF) diet has become one of the common dietary styles worldwide, resulting in metabolic health problems such as fatty liver, which is characterized by an extravagant aggregation of triglycerides (TGs) in hepatocytes (hepatic steatosis). HF diet-associated fatty liver (also known as nonalcoholic fatty liver disease, NAFLD) is usually associated with hyperlipidemia, overweight/or obesity, and type 2 diabetes and is therefore considered the hepatic component of metabolic syndrome. Simple hepatic steatosis can progress to nonalcoholic steatohepatitis (NASH) and liver fibrosis, eventually causing cirrhosis and/or liver cancer without any appropriate intervention [1]. Therefore, NAFLD has attracted widespread attention due to the increase in its prevalence from 20 to 41% worldwide [2], signifying its prevention and treatment as a strong public interest. However, there are currently no pharmaceutical interventions approved for the treatment of NAFLD [3]. Lifestyle changes, especially daily dietary management, are considered to be an effective strategy [4, 5].

The primary factor that triggers hepatic steatosis is known to be lipid overload. The accumulation of excessive fat causes hepatic lipotoxicity, which induces liver cells to release proinflammatory cytokines, triggers oxidative stress, and activates hepatic stellate cells, ultimately leading to hepatic inflammatory injury. Improvement in lipid metabolism is an effective and basic strategy for treating NAFLD [6].

Quinoa is a pseudocereal and abundant in proximate nutrients such as carbohydrates, high-quality proteins, dietary fiber, and microelements [7]. In particular, quinoa is a rich source of bioactive phytochemicals such as polyphenols, flavonoids, rutins, and saponins [8, 9]. Among these phytochemicals, phenolic compounds and saponins in quinoa are phytochemicals that have attracted considerable attention from researchers [10]. A recent study reported that these phytochemicals of quinoa had potentially beneficial effects on human health. For example, Yao et al. [11] found that quinoa saponins inhibited the release of inflammatory cytokines, including tumor necrosis factor (TNF)-α and interleukin-6, in lipopolysaccharide-induced RAW264.7 cells and suggested that quinoa saponins could be used as functional food components to prevent and treat inflammation. Other studies reported that phytochemicals in quinoa lowered the risk of oxidative stress-related diseases, e.g., cancer, cardiovascular diseases, diabetes, and obesity [12]. In particular, a clinical trial showed that the consumption of 50 g quinoa/day lowered serum triglycerides (TGs) in overweight and obese participants and reduced the prevalence of metabolic syndrome [13]. These beneficial effects of quinoa implied that it may prevent the progression of NAFLD. In fact, Mohamed et al. [14] reported that quinoa powder improved biochemical parameters to different degrees in NAFLD rats. Similarly, Noratto et al. [15] demonstrated that quinoa intake reduced plasma and liver cholesterol, decreased obesity-associated inflammation, and prevented hepatic steatosis in obese db/db (Leprdb/db) mice. However, the action of quinoa on lipid metabolism in NAFLD and its related molecular mechanism still need to be elucidated. In the present study, the effects of different amounts of quinoa intake on the metabolic profile of rats fed a HF diet were explored using nontarget metabolomics, and the transcriptome expression profile was also investigated using RNA-Seq.

## Materials and methods

### Materials

Quinoa is grown in the mountain dryland areas of the Qinghai-Tibet Plateau (at an altitude of 4000 m) and was provided by Qinghai Sanjiang Wotu Ecology Agriculture Technology Co., Ltd. (Qinghai, China).

The assay kits for SOD (superoxide dismutase) (A001-3-2) and GSH-PX (glutathione peroxidase) (A005-1-2) activity and the levels of MDA (malondialdehyde) (A003-1-2), GSH (glutathione) (A006-1-1), TG (A110-2-1), and TC (total cholesterol) (A111-2-1) were purchased from Jiancheng Biological (Nanjing, China). All other chemical reagents used were purchased from Sinopharm (Shanghai, China).

### Animals and experimental design

Male Sprague–Dawley (SD) rats (specific pathogen-free [SPF] grade, 4 w, 100 ± 10 g) were provided by Vital River Laboratory Animal Technology Co., Ltd. (Beijing, China). Animals were maintained in a ventilated rack system. Food and water were provided ad libitum throughout the entire study. After a week of adaptive feeding, the rats were randomly allocated to the following four groups containing animals with similar mean body weights (BWs): a normal chow diet control group (NC, *n* = 7), a high-fat diet group (HF, *n* = 7), a low-dose quinoa diet group (HF + LQ, *n* = 7), and a high-dose quinoa diet group (HF + HQ, *n* = 7). The rats in the NC group were fed a standard diet, and those in the other three groups were fed a HF diet. Simultaneously, low-dose quinoa feed (HF + LQ) was added to 9% quinoa (equivalent to 100 g of daily human intake), and high-dose quinoa feed (HF + HQ) was added to 27% quinoa (equivalent to 300 g of daily human intake). The special feed was manufactured by FBSH Biotechnology Co., Ltd (Shanghai, China).

All rats were observed daily, and the BWs of all animals were recorded once a week. The experimental conditions and procedures were approved by the Shanghai Jiao Tong University Institutional Animal Care and Use Committee (A2018070).

### Sample collection

After 12 weeks of the intervention, the rats were fasted overnight, weighed, and anesthetized with 2% sodium pentobarbital (0.2 mL/100 g) according to the recommendations for experimental animals. Blood samples were collected from the abdominal aorta, and serum was isolated by low-speed centrifugation (3509×g, 10 min, 4 °C). Livers were rapidly removed and weighed. Fresh liver samples from the same lobe and similar site in each rat were immediately fixed in 4% paraformaldehyde, and the other tissues were flash-frozen in liquid nitrogen. All samples were kept at − 80 °C until further analysis.

### Histopathological analysis

The fixed liver tissues were processed and embedded in paraffin for hematoxylin–eosin (H&E) staining. The pathological sections were observed and analyzed under an optical microscope (Olympus Soft Imaging Solutions GmbH, Münster, Germany).

### Determination of hepatic TG and TC

Briefly, fresh liver tissues (50 mg of each sample) were extracted and homogenized using a tenfold volume of ethanol. Then, 10 μL of supernatant was taken for determination after centrifugation. The levels of TG (GPO-PAP method) and TC (GPO-PAP method) in hepatic tissues were evaluated following the manufacturer’s instructions.

### Analysis of antioxidative parameters

The activities of serum SOD and GSH-Px and the levels of GSH and MDA were evaluated according to the manufacturer’s instructions. Briefly, 50 μL, 30 μL and 25 μL of serum were used for the determination of SOD activity as well as the GSH and MDA levels. For the measurement of GSH-PX, 100 μL of serum was used after 2 times of dilutions.

### Analysis of chemical composition of rat feed

The crude protein content and fat content were determined using the Kjeldahl method (AOAC International (AOAC) 2001.11) and the ether extract method (AOAC, 920.39), respectively. The content of crude fiber was analyzed using an AOAC method (AOAC, 962.09). The ash (AOAC 942.05) and moisture (AOAC 2001.12) contents were measured using the gravimetric method. The content of carbohydrates in animal feeding was calculated according to the following method: 100 − content of moisture, ash, fat, protein and crude fiber.

### Differential metabolite analysis in serum and hepatic tissue using UPLC-QTOF-MS^E^ (ultraperformance liquid chromatography-quadrupole time-of-flight mass spectrometry)

For liver tissue, 50 mg of freeze-dried liver powder was extracted with 500 µL of methanol/acetonitrile/water (1/1/1, v/v/v) by a homogenizer and incubated at − 20 °C for 2 h. The supernatant was collected for analysis after centrifugation (12,000 rpm, 4 °C, 20 min).

For serum, 200 µL of methanol/acetonitrile (1/1, v/v) was added to 50 µL of serum, extracted at 4 °C for 2 h, and vortexed every other 15 min during the extraction. The supernatant (100 µL) was collected for analysis after centrifugation (12,000 rpm, 4 °C, 25 min).

Chromatography was performed with an ACQUITY UPLC BEH C18 chromatographic column (100 × 2.1 mm, 1.7 µm; Waters Co., Massachusetts, USA) and maintained at 45 °C. The gradient elution program for mobile phases A (0.1% formic acid–water) and B (0.1% formic acid–acetonitrile) was as follows: 0–1 min, 5–20% B; 1–2.5 min, 20–40% B; 2.5–9 min, 40–100% B; 9–12 min, 100% B; 12–12.5 min, 100–5% B; 12.5–14.5 min, 5% B; flow rate, 0.4 mL/min and injection volume: 1 µL.

The metabolites were identified using the UPLC-QTOF-MS^E^ system (Acquity UPLC I-class & Vion IMS Qtof MS, Waters Co. USA) as follows. The ion source was operated in positive and negative electrospray ionization (ESI) modes. The MS^E^ scan mode alternated between low-energy (CE 4 eV) and high-energy (CE 20–45 eV) MS^E^ modes. The scanning range was 50 to 1000 amu, and the scanning speed was maintained at 0.2 s/scan; capillary voltages were 1 kV (negative mode) and 1.5 kV (positive mode), cone voltage was 40 V, source bias was 60 V, source temperature was 115 °C, desolvation gas temperature was 450 °C, and the flow rate of the desolvation gas was 900 L/h, along with a cone gas flow rate of 50 L/h. The locking mass calibration Tyr-Gly-Phe-Leu (leucine–encephalin; 250 pg/µL) was used as the lock mass (*m/z*: 554.2620) for real-time calibration.

To ensure the consistency of the analytical system, pooled quality control (QC) samples were made by mixing all the samples. Ten QC samples were injected to equilibrate the system before analysis. To evaluate the stability of the system, a QC sample was injected after each 10 sample injections during the analysis process.

### RNA-Seq

Total RNA was extracted from fresh liver tissues (50 mg of each sample) using TRIzol reagent (Invitrogen, USA). cDNA libraries were constructed, sequenced, and analyzed by Huayou Biological Technology Co., Ltd. (Shanghai, China).

### RNA extraction and quantitative real-time polymerase chain reaction (PCR) verification

First-strand cDNA was synthesized using the Prime-Script RT reagent kit (Takara Bio Inc., China). SYBR Premix Ex TaqII (Takara Bio Inc., Shanghai, China) was used in quantitative real-time polymerase chain reaction (qRT-PCR). β-Actin was used as an endogenous reference for mRNA expression. The comparative 2^−ΔΔCt^ method was used to calculate the relative fold-change of target gene expression. The sequences of the primers in this study are shown in Additional file 1: Table S2.

### Data processing and statistical analysis

Chromatographic and MS data (retention time, accurate mass, ion intensity, MS^2^ fragment) were collected by UNIFI 1.8.1 (Waters Co., USA) and identified by Progenesis QI v2.3 (Waters Co., USA) based on an online database (MS/MS spectral database, HMDB [http://www.hmdb.ca/], LIPID MAPS [http://www.lipidmaps.org] and Metlin: https://metlin.scripps.edu/). Partial least squares discrimination analysis (PLS-DA) was performed to visually discriminate among the samples of different treatment groups. The differential metabolites were searched based on the variable importance in the projection (VIP > 1), the smallest coefficient of variation (min. CV < 30%), and the p value (p < 0.05).

All parameters are expressed as the mean ± standard deviation (SD). The results were statistically analyzed by one-way analysis of variance (ANOVA) followed by the least significant difference (LSD) multiple comparison test. The criterion for significance was p < 0.05.

## Results

### Effect of quinoa diet on food intake and body weight of rats fed a high-fat diet

**Fig. 1:**
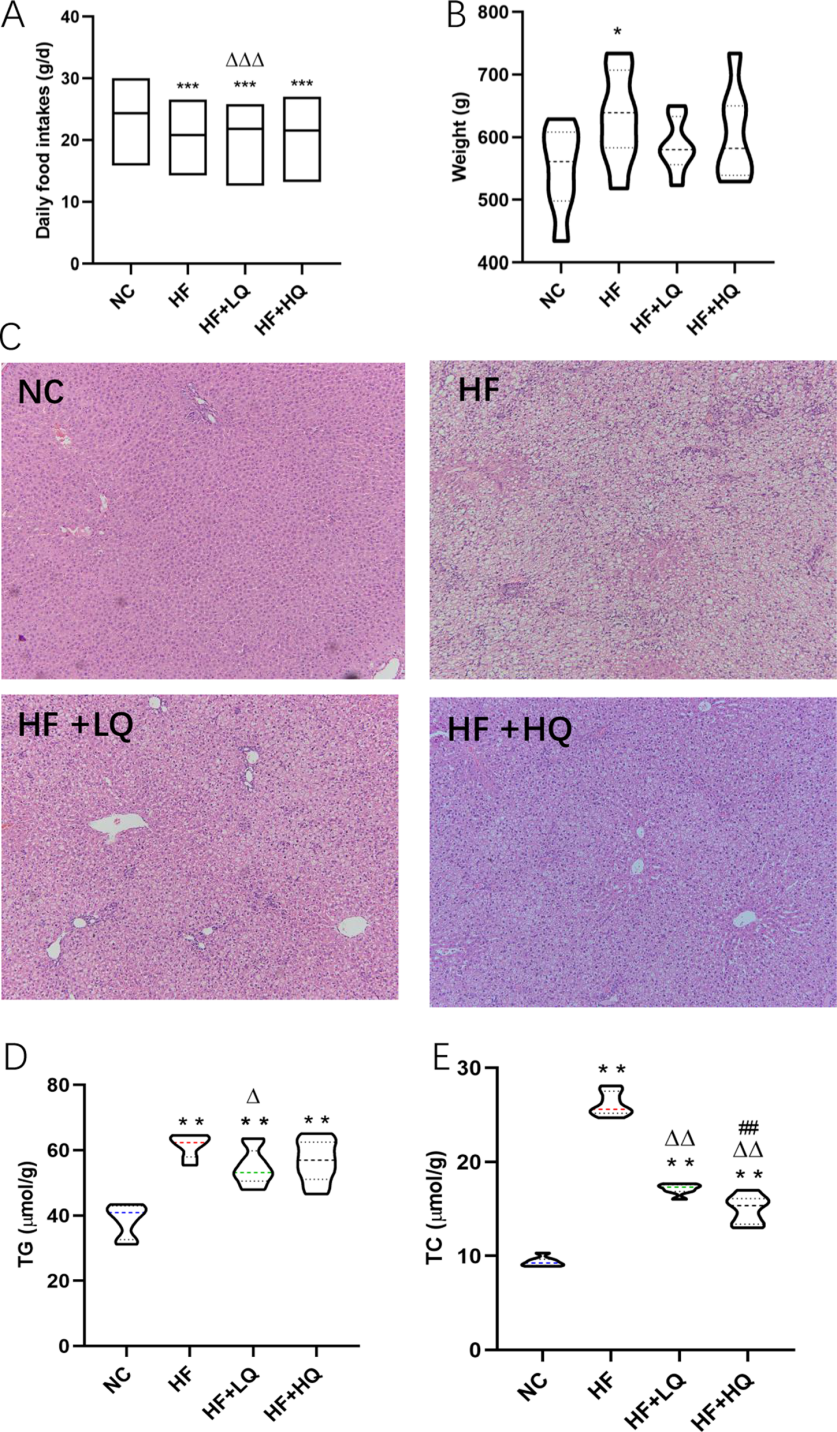
Effects of quinoa on daily food intake, weight and hepatic steatosis histology changes of rats fed a high-fat diet. **A** Daily food intake. **B** Weight. **C** Hepatic histology. **D** Hepatic TG content. **E** Hepatic TC content. NC: normal chow diet control group (*n* = 7); HF: a high-fat diet group (*n* = 7); HF + LQ: low-dose quinoa diet group (*n* = 7); HF + HQ: a high-dose quinoa diet group (*n* = 7). The rats in the NC group were fed a standard diet, and those in the other three groups were fed a HF diet. Simultaneously, low-dose quinoa feed (HF + LQ) was added to 9% quinoa (equivalent to 100 g of daily human intake), and high-dose quinoa feed (HF + HQ) was added to 27% quinoa (equivalent to 300 g of daily human intake). *p < 0.05 vs. the NC group, **p < 0.01 vs. the NC group; ^Δ^*p* < 0.05 vs. the HF group, ^ΔΔ^*p* < 0.01 vs. the HF group; ^#^*p* < 0.05 vs. the HF + LQ group; ^##^*p* < 0.01 vs. the HF + LQ group. The bars show the mean values with standard deviations

The macronutrient composition and energy of feed for SD rats in each group are shown in Additional file 1: Table S1. The average food intake of the NC group was 24 g/d, remarkably higher (*p* < 0.01) than the average food intake of the HF (21 g/d), HF + LQ (22 g/d) and HF + HQ (21 g/d) groups (Fig. 1A). The corresponding food calories were 355 kJ/d, 421 kJ/d, 433 kJ/d and 404 kJ/d for the NC and three HF diet groups, suggesting that an HF diet reduced food intake due to higher calories than normal feed. However, the intake of feed containing quinoa, especially low-dose quinoa, was significantly higher than the intake of feed of the HF diet alone (*p* < 0.01), implying that quinoa may improve the appetite of rats.

The average body weight (BW) of rats in the HF diet alone group was 630 g at the end of the experiment, which was significantly higher than the average BW of rats in the NC group (550 g) (*p* < 0.01), whereas the average BWs were 586 g and 604 g in the HF + LQ and HL + HQ groups, respectively. There was no significant difference compared with the NC group and the two quinoa feeding groups (Fig. 1B).

### Effect of quinoa diet on hepatic pathological changes and lipid content of rats fed a HF diet

A long-term HF diet can cause lipid deposition in the liver, resulting in insulin resistance and further inducing metabolic disturbance to form a vicious cycle. Hepatic histopathological changes were observed in rats fed an HF diet, and HE-stained pathological sections of hepatic tissues are shown in Fig. 1C. In the NC group, the hepatic lobules were clear, and hepatocytes were intact without fat accumulation. However, there were large lipid droplets, balloon-like degeneration, partial necrosis, multiple inflammatory aggregations, and severe steatosis of hepatocytes in the hepatic tissue of rats in the HF group. Hepatic intracellular lipid drop accumulation in the rats of the two quinoa diet groups was reduced compared with the lipid drop accumulation of those in the HF group. The analysis of TG and TC levels in the hepatic tissue of rats in each group supported the histopathology results. The TC and TG contents of the HF, HF + LQ, and HF + HQ groups significantly increased compared with the TC and TG contents of the NC group (*p* < 0.01), whereas the TG (*p* < 0.05) and TC (*p* < 0.01) contents in the hepatic tissue of rats in the HF + LQ and TC content (*p* < 0.01) in the hepatic tissue of rats in the HF + HQ groups were significantly lower than the TG and TC contents of the HF group (Fig. 1D, E).

### Effects of a quinoa diet on serum antioxidative parameters of rats fed a high-fat diet

**Fig. 2:**
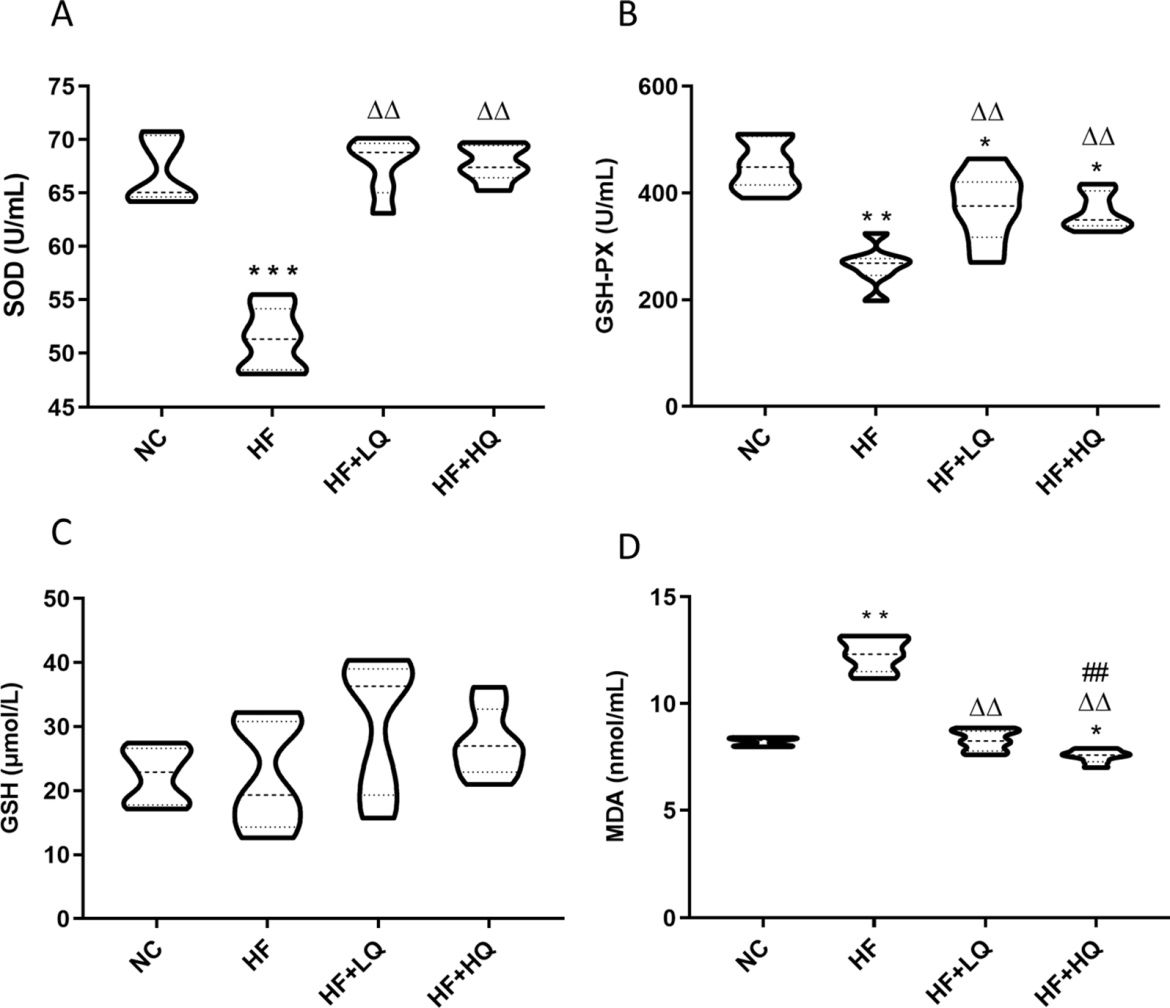
Effects of quinoa on serum antioxidative parameters of rats fed a high-fat diet. **A** SOD activity. **B** GSH-PX activity. **C** GSH level. **D** MDA level. NC: normal chow diet control group (*n* = 7); HF: a high-fat diet group (*n* = 7); HF + LQ: low-dose quinoa diet group (*n* = 7); HF + HQ: a high-dose quinoa diet group (*n* = 7). The rats in the NC group were fed a standard diet, and the rats in the other three groups were fed an HF diet. Simultaneously, low-dose quinoa feed (HF + LQ) was added to 9% quinoa (equivalent to 100 g of daily human intake), and high-dose quinoa feed (HF + HQ) was added to 27% quinoa (equivalent to 300 g of daily human intake). *p < 0.05 vs. the NC group, **p < 0.01 vs. the NC group; ^Δ^*p* < 0.05 vs. the HF group, ^ΔΔ^*p* < 0.01 vs. the HF group; ^#^*p* < 0.05 vs. the HF + LQ group; ^##^*p* < 0.01 vs. the HF + LQ group. The bars show the mean values with standard deviations

The serum activities of SOD and GSH-Px and the levels of GSH and MDA in rats of each group are displayed in Fig. 2A–D. The SOD and GSH-Px activities of rats fed an HF diet alone were reduced by 23.8% and 41.3% compared with those in the NC group (*p* < 0.01), while the SOD and GSH-PX activities of rats in the HF + LQ and HF + HQ groups were distinctly higher than those of rats in the HF group (*p* < 0.01) (Fig. 2A, B). The GSH levels of rats in the HF groups were lower than the GSH levels of rats in the NC, HF + LQ, and HF + HQ groups, and the GSH levels of rats in the HF + LQ and HF + HQ groups were higher than the GSH levels of rats in NC and HF groups, but there were no significant differences among the four groups (Fig. 2C).

MDA is a representative harmful lipid peroxide. The MDA levels in the rats of the HF group were significantly increased by 50% compared to those in the NC group (*p* < 0.01), whereas the quinoa diet inhibited the increase in MDA levels compared with the high fat diet alone group (*p* < 0.01) (Fig. 2D). High intake of quinoa further inhibited the production of MDA compared with low quinoa intake (*p* < 0.01), probably due to higher levels of antioxidative phytochemicals. The above results suggested that quinoa effectively ameliorated the oxidative stress induced by an HF diet.

### Effects of quinoa diet on serum metabolites of rats fed a high-fat diet

**Fig. 3:**
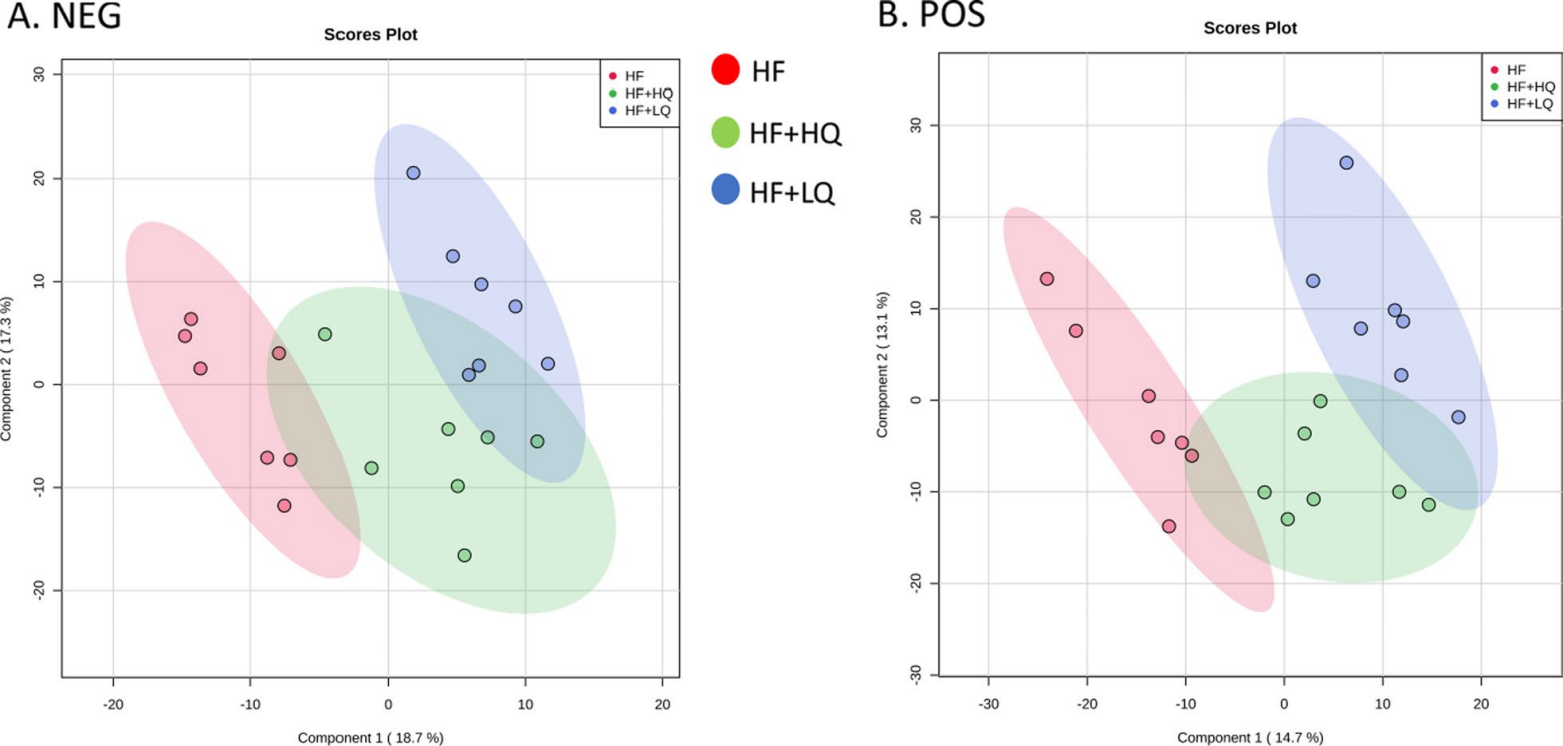

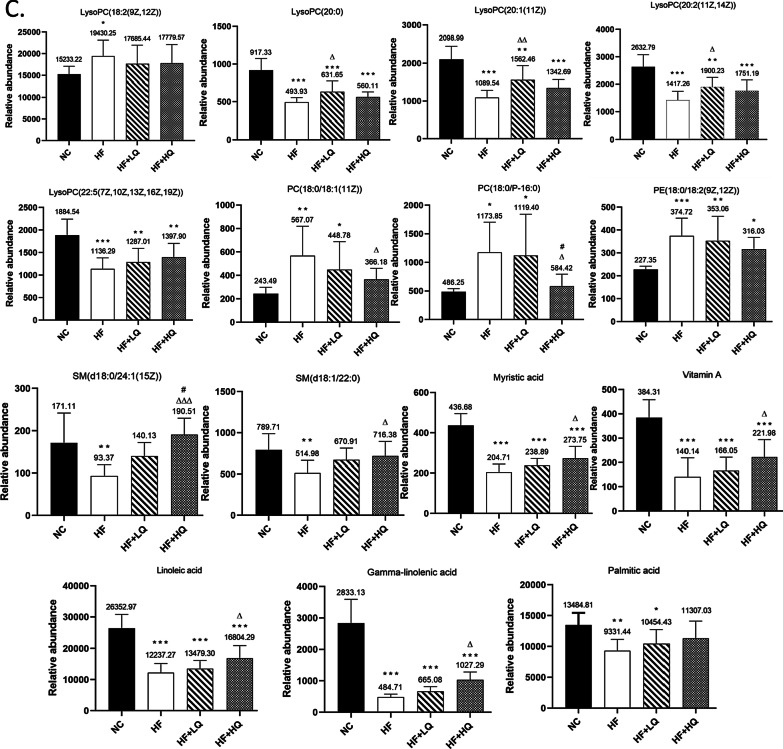
Untargeted serum metabolomics analysis of rats fed a high-fat diet. **A** and **B** PLS-DA (partial least squares discrimination analysis) score plot under negative and positive ion modes for serum metabolites obtained by UPLC-QTOF-MS^E^. **C** Partial differential metabolites in the serum. NC: normal chow diet control group (*n* = 7); HF: a high-fat diet group (*n* = 7); HF + LQ: low-dose quinoa diet group (*n* = 7); HF + HQ: a high-dose quinoa diet group (*n* = 7). The rats in the NC group were fed a standard diet, and those in the other three groups were fed a HF diet. Simultaneously, low-dose quinoa feed (HF + LQ) was added to 9% quinoa (equivalent to 100 g of daily human intake), and high-dose quinoa feed (HF + HQ) was added to 27% quinoa (equivalent to 300 g of daily human intake). *p < 0.05 vs. the NC group, **p < 0.01 vs. the NC group; ^Δ^*p* < 0.05 vs. the HF group, ^ΔΔ^*p* < 0.01 vs. the HF group; ^#^*p* < 0.05 vs. the HF + LQ group; ^##^*p* < 0.01 vs. the HF + LQ group. The bars show the mean values with standard deviations

Given the improvements by quinoa on hepatic steatosis and oxidative stress in HF diet-fed rats, we further investigated the serum metabolites using untargeted metabolomics analysis. The score plots of each group showed distinct clustering, and the serum metabolite profile of rats in the quinoa diet group was different from the serum metabolite profile of rats in the high fat diet alone groups (Fig. 3A and B).

A total of 1863 metabolites (ESI−, 691; ESI+, 1172) were detected by UPLC-Q-TOF-MS^E^, and 45 differential serum metabolites are shown in Table 1. The differential metabolites included mainly the lipids related metabolites PC (phosphatidylcholine), LysoPC (lysophosphatidylcholine), and PE (phosphatidylethanolamine).

**Table 1:** Identification of differential metabolites in the serum of rats in each group

Component name	MS (*m/z*)	Observed RT (min)	Mass error (ppm)	Formula	Adducts	VIP	Trend		
							NC/HF	HF + LQ/HF	HF + HQ/HF
SM(d18:0/24:1(15Z))	837.6820	10.45	− 0.4	C_47_H_95_N_2_O_6_P	M + H, M + Na	1.4	↑	↑	↑
SM(d18:1/22:0)	809.6501	10.08	− 0.5	C_45_H_91_N_2_O_6_P	M + H, M + Na	2.5	↑	↑	↑
SM(d18:1/16:0)	703.5745	8.94	− 2.4	C_39_H_79_N_2_O_6_P	M + H− H_2_O, M + H	4.7	↑	↑	↓
PC(16:0/20:3(8Z,11Z,14Z))	784.5844	8.95	− 0.8	C_44_H_82_NO_8_P	M + H, M + Na	4.0	↓	↑	↑
PC(16:0/22:5(7Z,10Z,13Z,16Z,19Z))	852.5746	8.77	− 1.8	C_46_H_82_NO_8_P	M + FA-H	1.0	↑	↑	↓
PC(16:1(9Z)/20:4(8Z,11Z,14Z,17Z))	780.5532	8.49	− 0.7	C_44_H_78_NO_8_P	M + H, M + Na	1.6	↑	↓	↑
PC(18:1(9Z)/18:1(9Z))	808.5833	8.78	0.8	C_44_H_84_NO_8_P	M + Na	2.7	↑	↑	↑
PC(18:0/18:1(9Z))	788.6158	9.52	− 0.7	C_44_H_86_NO_8_P	M + H	3.0	↓	↓	↓
PC(18:0/16:0)	762.6001	9.54	− 0.8	C_42_H_84_NO_8_P	M + H	1.0	↓	↓	↓
PC(18:0/22:6(4Z,7Z,10Z,13Z,16Z,19Z))	834.5998	8.98	− 1.1	C_48_H_84_NO_8_P	M + H, M + Na	5.4	↑	↓	↓
PC(18:0/20:3(5Z,8Z,11Z))	812.6154	9.27	− 1.2	C_46_H_86_NO_8_P	M + H	3.1	↓	↓	↓
PC(18:0/18:2(9Z,12Z))	786.6002	9.22	− 0.7	C_44_H_84_NO_8_P	M + H	4.8	↓	↓	↓
PC(18:2(9Z,12Z)/20:4(5Z,8Z,11Z,14Z))	828.5507	8.52	− 0.9	C_46_H_80_NO_8_P	M + H, M + Na	3.5	↑	↓	↑
PC(18:0/P-16:0)	768.5872	9.40	− 0.7	C_42_H_84_NO_7_P	M + H, M + Na	3.2	↓	↓	↓
PC(18:0/18:1(11Z))	832.6059	9.53	− 1.9	C_44_H_86_NO_8_P	M + FA-H	1.6	↓	↓	↓
PC(20:3(5Z,8Z,11Z)/16:0)	806.5687	8.67	2.1	C_44_H_82_NO_8_P	M + Na	4.9	↑	↓	↓
PC(20:2(11Z,14Z)/18:0)	836.6147	9.09	0.9	C_46_H_88_NO_8_P	M + Na	1.1	↑	↑	↓
PC(20:4(5Z,8Z,11Z,14Z)/22:6(4Z,7Z,10Z,13Z,16Z,19Z))	854.5694	8.33	0.0	C_50_H80NO8P	M + H, M + Na	1.1	↑	↓	↓
PE(18:0/18:2(9Z,12Z))	742.5386	8.90	− 0.8	C_41_H_78_NO_8_P	M-H	1.0	↓	↓	↓
PE(22:5(4Z,7Z,10Z,13Z,16Z)/18:1(11Z))	790.5392	8.68	0.0	C_45_H_78_NO_8_P	M-H	1.1	↑	↓	↓
LysoPC(16:0)	496.3397	6.03	− 0.2	C_24_H_50_NO_7_P	M + H-H_2_O, M + H, 2M + H	6.8	↑	↑	↓
LysoPC(15:0)	482.3238	5.79	− 0.7	C_23_H_48_NO_7_P	M + H-H_2_O, M + H, M + Na	2.2	↑	↑	↓
LysoPC(14:0)	468.3081	5.32	− 0.4	C_22_H_46_NO_7_P	M + H-H_2_O, M + H, M + Na	2.2	↑	↑	↑
LysoPC(16:1(9Z))	494.3238	5.52	− 0.7	C_24_H_48_NO_7_P	M + H-H_2_O, M + H, M + Na	3.8	↑	↑	↑
LysoPC(20:0)	552.4022	7.51	1.0	C_28_H_58_NO_7_P	M + H-H_2_O, M + H, M + Na	1.4	↑	↑	↑
LysoPC(20:2(11Z,14Z))	548.3707	6.51	− 0.6	C_28_H_54_NO_7_P	M + H, M + Na	2.4	↑	↑	↑
LysoPC(20:1(11Z))	550.3861	6.99	− 1.1	C_28_H_56_NO_7_P	M + H	2.7	↑	↑	↑
LysoPC(20:4(8Z,11Z,14Z,17Z))	544.3394	5.55	− 0.7	C_28_H_50_NO_7_P	M + H-H_2_O, M + H, M + Na	5.1	↑	↓	↑
LysoPC(22:5(4Z,7Z,10Z,13Z,16Z))	570.3529	6.07	− 4.4	C_30_H_52_NO_7_P	M + H, M + Na	1.8	↓	↓	↑
LysoPC(20:3(5Z,8Z,11Z))	546.3551	6.04	− 0.5	C_28_H_52_NO_7_P	M + H-H_2_O, M + H	5.6	↓	↑	↑
LysoPC(22:5(7Z,10Z,13Z,16Z,19Z))	570.3548	5.85	− 1.1	C_30_H_52_NO_7_P	M + H-H_2_O, M + H, M + Na	2.0	↑	↑	↑
LysoPC(22:6(4Z,7Z,10Z,13Z,16Z,19Z))	568.3394	5.60	− 0.7	C_30_H_50_NO_7_P	M + H-H_2_O, M + H, M + Na	6.6	↑	↓	↓
LysoPC(P-18:0)	508.3756	6.68	− 1.1	C_26_H_54_NO_6_P	M + H, M + Na	3.7	↑	↑	↓
LysoPC(18:2(9Z,12Z))	564.3301	5.78	− 1.2	C_26_H_50_NO_7_P	M + FA-H	5.5	↓	↓	↓
LysoPC(P-16:0)	524.3352	6.49	− 1.2	C_24_H_50_NO_6_P	M + FA-H	1.0	↑	↑	↓
Docosahexaenoic acid	327.2326	6.62	− 1.0	C_22_H_32_O_2_	M-H	6.6	↑	↓	↓
Myristic acid	227.2011	6.57	− 2.3	C_14_H_28_O_2_	M-H	1.3	↑	↑	↑
Linoleic acid	279.2328	6.86	− 0.6	C_18_H_32_O_2_	M-H	7.0	↑	↑	↑
Palmitic acid	255.2326	7.27	− 1.3	C_16_H_32_O_2_	M-H	5.4	↑	↑	↑
Arachidonic acid	303.2328	6.75	− 1.1	C_20_H_32_O_2_	M-H_2_O-H, M-H	8.1	↑	↑	↑
Taurocholic acid	496.2733	3.31	− 1.0	C_26_H_45_NO_7_S	M-H2O-H	2.9	↑	↓	↓
Gamma-linolenic acid	277.2169	6.40	− 1.4	C_18_H_30_O_2_	M-H	3.1	↑	↑	↑
Eicosapentaenoic acid	303.2315	5.28	− 1.1	C_20_H_30_O_2_	M + H-H_2_O, M + H, M + Na, 2M + H	5.8	↑	↑	↑
Sphingosine	282.2788	4.61	− 1.2	C_18_H_37_NO_2_	M + H-H_2_O, M + H	1.1	↓	↓	↓
Vitamin A	287.2366	7.31	− 1.1	C_20_H_30_O	M + H	1.1	↑	↑	↑

Specifically, in comparison with the HF group, the low amount of quinoa (HF + LQ), significantly increased the levels of serum LysoPC (20:0, *p* < 0.05; 20:1(11Z), *p* < 0.01; 20:2(11Z, 14Z), *p* < 0.05); the high amount of quinoa (HF + HQ) increased the levels of SM (sphingomyelin, 18:0/24:1 (15Z); d18:1/22:0, *p* < 0.01), gamma-linolenic acid, vitamin A, linoleic acid, myristic acid, and decreased the levels of PC (18:0/18:1 (11Z); 18:0/P-16:0) (*p* < 0.05) (Fig. 3C).

### Effects of quinoa diet on hepatic metabolites of rats fed a high-fat diet

**Fig. 4:**
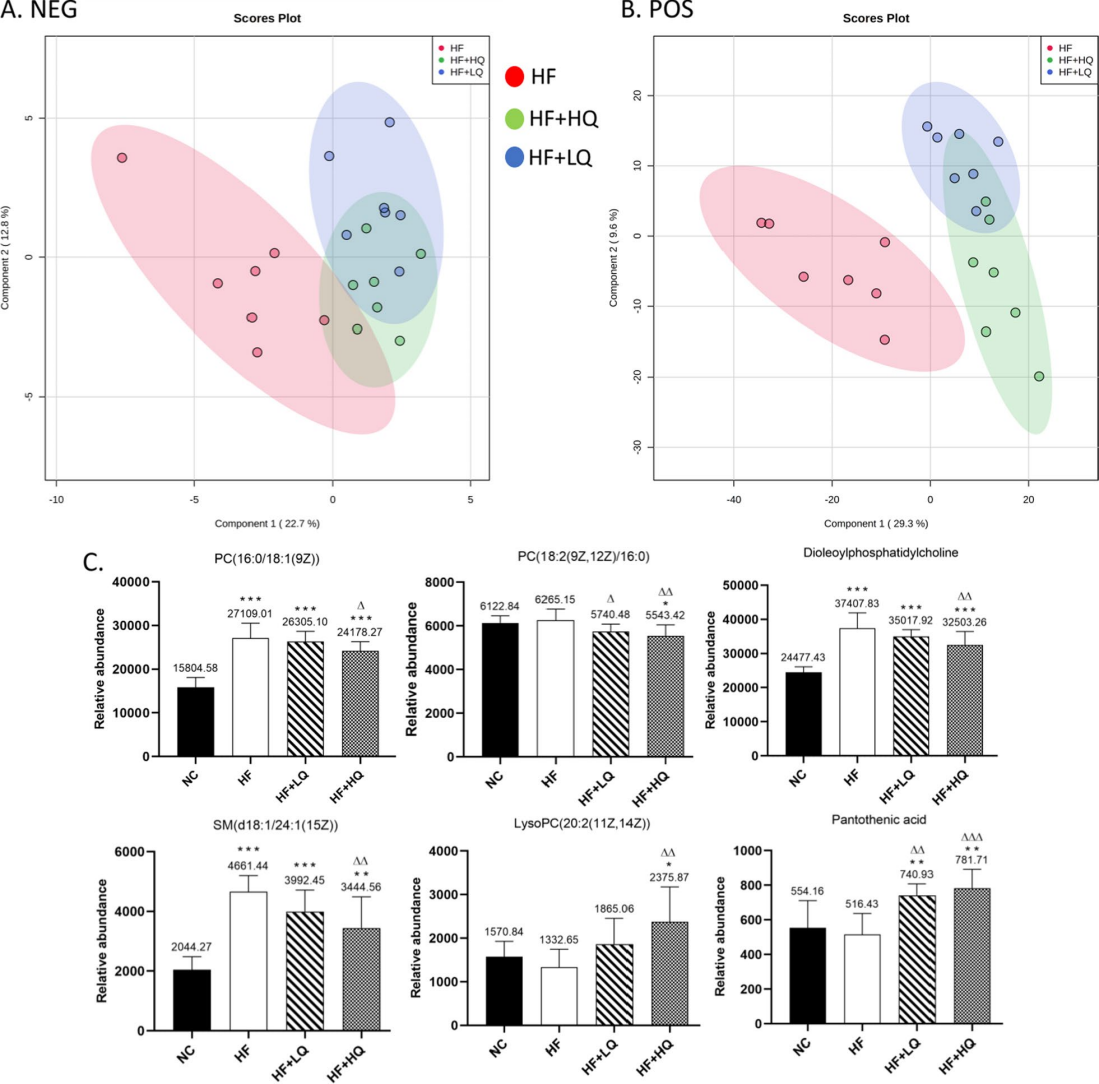
Untargeted hepatic metabolomics analysis of rats fed a high-fat diet. **A** and **B** PLS-DA score plot under negative and positive ion modes for the hepatic metabolites obtained by UPLC-QTOF-MS^E^. **C** Partial differential metabolites in the liver. NC: normal chow diet control group (*n* = 7); HF: a high-fat diet group (*n* = 7); HF + LQ: low-dose quinoa diet group (*n* = 7); HF + HQ: a high-dose quinoa diet group (*n* = 7). The rats in the NC group were fed a standard diet, and those in the other three groups were fed a HF diet. Simultaneously, low-dose quinoa feed (HF + LQ) was added to 9% quinoa (equivalent to 100 g of daily human intake), and high-dose quinoa feed (HF + HQ) was added to 27% quinoa (equivalent to 300 g of daily human intake). *p < 0.05 vs. the NC group, **p < 0.01 vs. the NC group; ^Δ^*p* < 0.05 vs. the HF group, ^ΔΔ^*p* < 0.01 vs. the HF group; ^#^*p* < 0.05 vs. the HF + LQ group; ^##^*p* < 0.01 vs. the HF + LQ group. The bars show the mean values with standard deviations

Considering the important role of the liver in lipid metabolism, we further investigated hepatic metabolites. The score plots of each group exhibited distinct clustering, and the hepatic metabolite profile of rats in the quinoa diet group was different from the hepatic metabolite profile in the HF diet alone groups (Fig. 4A and B).

A total of 1295 (ESI−, 44; ESI+, 1251) hepatic metabolites were detected by UPLC-Q-TOF-MS^E^, and 22 differential hepatic metabolites are shown in Table 2. Specifically, the quinoa diet, especially the high amount of quinoa intake, significantly decreased the levels of PC (16:0/18:1 (9Z), *p* < 0.05; PC (18:2 (9Z, 12Z)/16:0), *p* < 0.01), dioleoylphosphatidylcholine (*p* < 0.01), and SM (d18:1/24:1(15Z)) (*p* < 0.01) and increased the levels of LysoPC (20:2(11Z, 14Z)) and pantothenic acid (Fig. 4C) (*p* < 0.05) in comparison with the HF group.

**Table 2:** Identification of differential metabolites in the liver of rats in each group

Component name	MS (*m/z*)	Observed RT (min)	Mass error (ppm)	Formula	Adducts	VIP	Trend		
							NC/HF	HF + LQ/HF	HF + HQ/HF
PC(20:4(5Z,8Z,11Z,14Z)/22:6(4Z,7Z,10Z,13Z,16Z,19Z))	854.5679	8.37	− 1.8	C_50_H_80_NO_8_P	M + H, M + Na	2.3	↑	↓	↓
PC(22:6(4Z,7Z,10Z,13Z,16Z,19Z)/16:1(9Z))	804.5511	8.41	− 3.4	C_46_H_78_NO_8_P	M + H, M + Na	3.2	↑	↑	↓
PC(20:5(5Z,8Z,11Z,14Z,17Z)/20:3(8Z,11Z,14Z))	830.5677	8.46	− 2.1	C_48_H_80_NO_8_P	M + H, M + Na	5.1	↑	↓	↓
PC(18:2(9Z,12Z)/16:0)	780.5509	8.94	− 0.6	C_42_H_80_NO_8_P	M + Na	1.5	↓	↓	↓
PC(16:0/18:1(9Z))	760.5842	9.20	− 1.1	C_42_H_82_NO_8_P	M + H	5.5	↓	↓	↓
Adenosine	268.1038	0.80	− 0.9	C_10_H_13_N_5_O_4_	M + H	1.0	↑	↓	↓
LysoPC(20:2(11Z,14Z))	548.3706	6.51	− 0.9	C_28_H_54_NO_7_P	M + H, M + Na	2.1	↑	↑	↑
LysoPC(15:0)	482.3241	7.02	− 0.1	C_23_H_48_NO_7_P	M + H, M + Na, 2M + H, 2M + Na	3.6	↓	↓	↑
SM(d18:1/24:1(15Z))	813.6857	10.05	1.6	C_47_H_93_N_2_O_6_P	M + H, M + Na	2.4	↓	↓	↓
Pantothenic acid	220.1178	1.67	− 0.8	C_9_H_17_NO_5_	M + H, M + Na	1.2	↑	↑	↑
Pyroglutamic acid	130.0498	1.15	− 0.6	C_5_H_7_NO_3_	M + H	1.1	↑	↓	↓
Pyrroline hydroxycarboxylic acid	130.0499	0.69	− 0.1	C_5_H_7_NO_3_	M + H	1.5	↑	↓	↓
Docosahexaenoic acid	329.2462	6.64	− 4.1	C_22_H_32_O_2_	M + H, M + NH4, M + Na	1.0	↓	↓	↓
Dipalmitoylphosphatidylcholine	734.5686	9.22	− 1.2	C_40_H_80_NO_8_P	M + H	2.3	↑	↓	↓
Glutathione	308.0906	0.77	− 1.5	C_10_H_17_N_3_O_6_S	M + H	8.8	↑	↑	↑
Oxidized glutathione	307.0833	0.69	0.0	C_20_H_32_N_6_O_12_S_2_	M + H, M + 2H	4.6	↑	↓	↓
Dioleoylphosphatidylcholine	786.5996	9.25	− 1.5	C_44_H_84_NO_8_P	M + H	6.0	↓	↓	↓
FAD	786.1630	1.70	− 1.8	C_27_H_33_N_9_O_15_P_2_	M + H	1.1	↑	↑	↓
Niacinamide	123.0553	0.67	0.1	C_6_H_6_N_2_O	M + H	1.2	↑	↓	↓
Pantetheine 4′-phosphate	359.1033	1.66	− 0.9	C_11_H_23_N_2_O_7_P_S_	M + H, M + Na, 2M + H, 2M + Na	2.7	↑	↑	↑
Inosine	267.0734	1.54	− 0.5	C_10_H_12_N_4_O_5_	M-H	3.9	↑	↓	↓
Glycerol 3-phosphate	171.0063	0.61	− 0.6	C_3_H_9_O_6_P	M-H	1.3	↑	↑	↑

PC: phosphatidylcholine; LysoPC: lysophosphatidylcholine; SM: sphingomyelin; FAD: flavin adenine dinucleotide

### Effects of quinoa diet on the hepatic mRNA expression profile of rats fed a high-fat diet

**Fig. 5:**
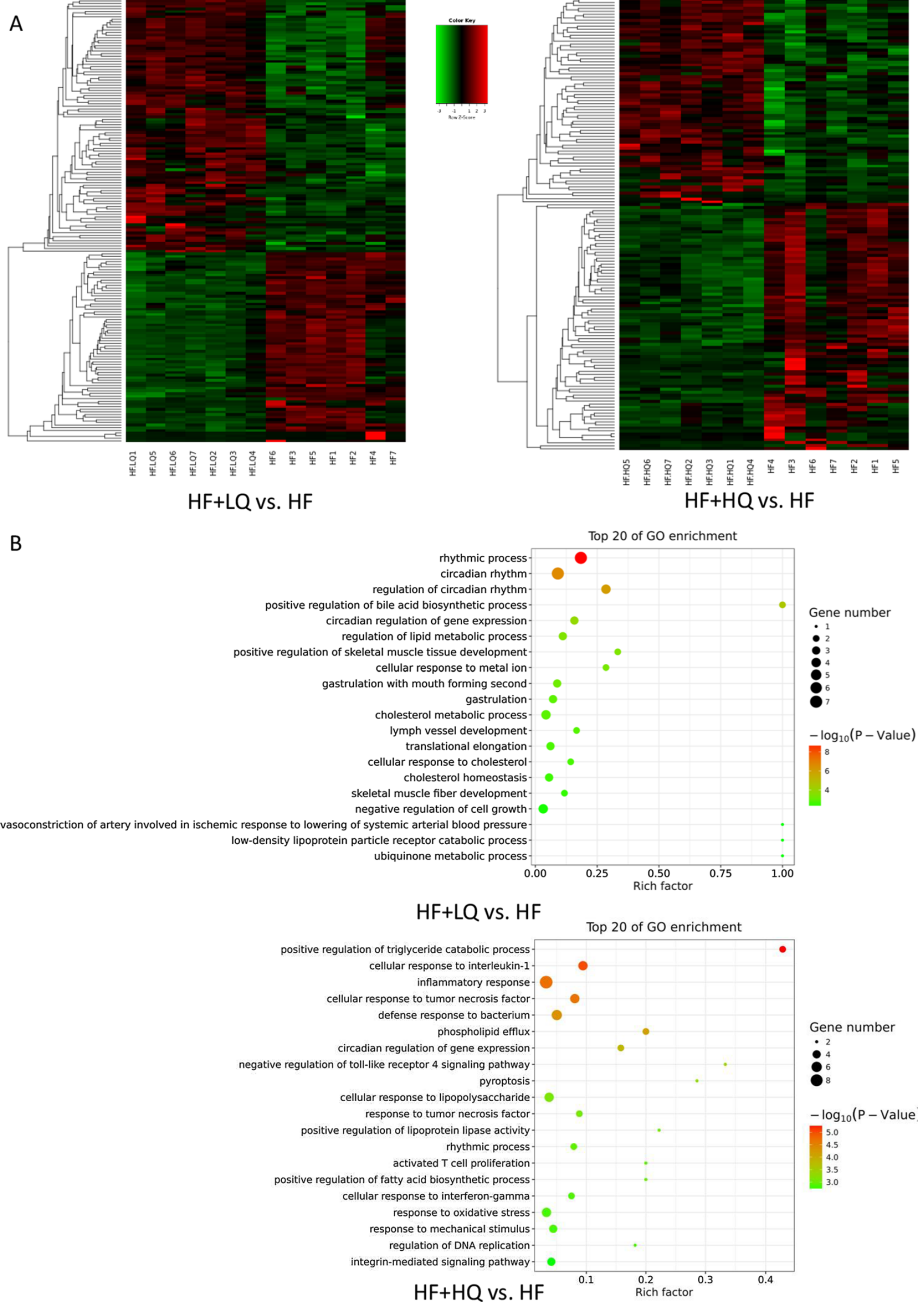

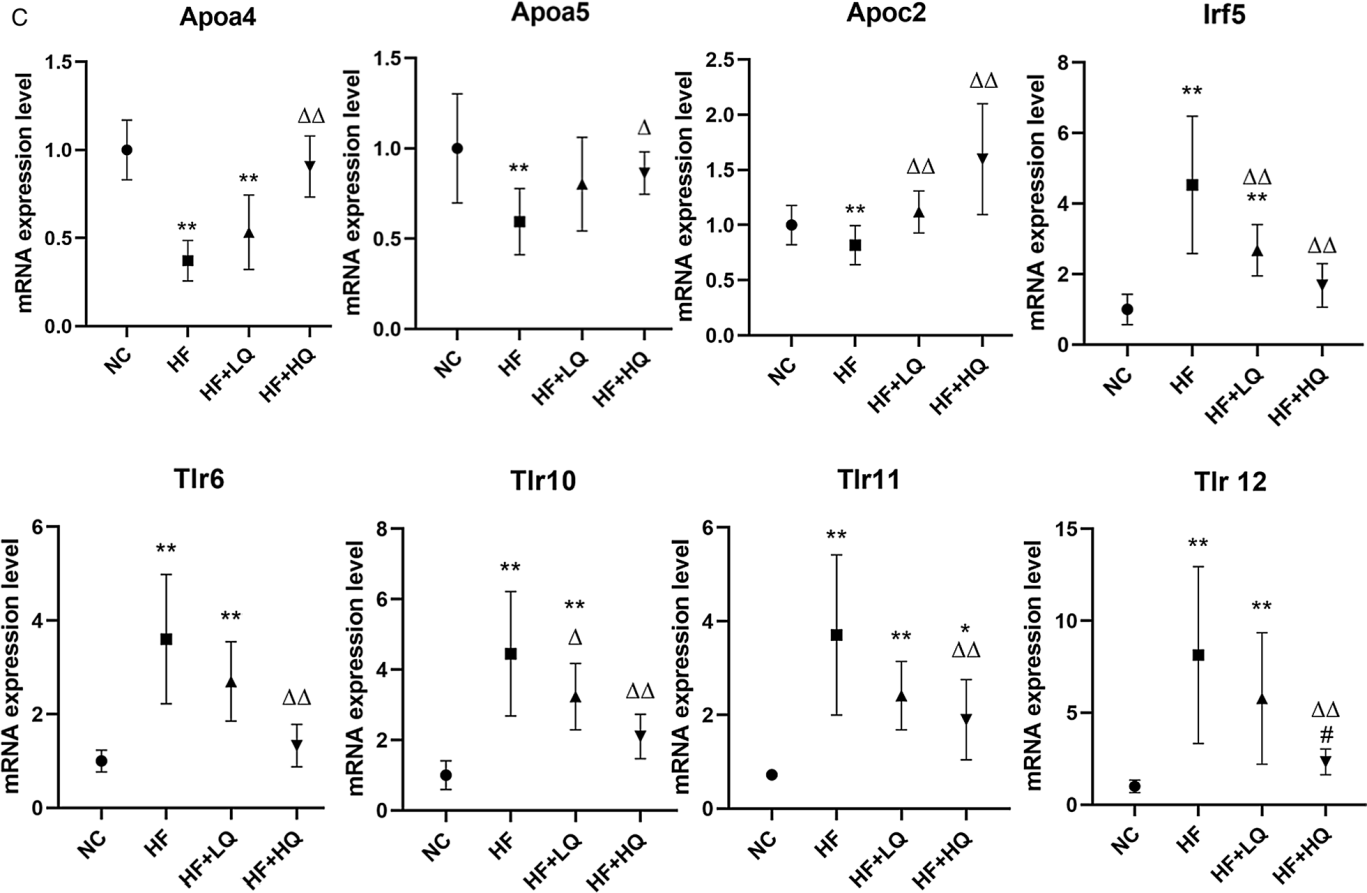
Effects of quinoa on the hepatic gene expression profile at the mRNA level in rats fed a high-fat diet. **A** Heatmap analysis; **B** GO analysis and clustering analysis; **C** Verification of partial gene mRNA expression levels using qRT-PCR. NC: normal chow diet control group (*n* = 7); HF: a high-fat diet group (*n* = 7); HF + LQ: low-dose quinoa diet group (*n* = 7); HF + HQ: a high-dose quinoa diet group (*n* = 7). The rats in the NC group were fed a standard diet, and those in the other groups were fed a HF diet. Simultaneously, low-dose quinoa feed (HF + LQ) was added to 9% quinoa (equivalent to 100 g of daily human intake), and high-dose quinoa feed (HF + HQ) was added to 27% quinoa (equivalent to 300 g of daily human intake)

To further investigate the mechanisms underlying the alleviating effects of quinoa on hepatic steatosis and its regulation of metabolism, we compared the transcriptome differences in the hepatic tissues between the HF + LQ, HF + HQ, and HF groups (Fig. 5). Heatmap analysis exhibited the opposite expression profile comparing the two quinoa diet intervention groups with the HF diet alone group (Fig. 5A). Notably, compared with the HF group, gene ontology (GO) analysis revealed that the lower amount of quinoa (HF + LQ) regulated mainly the genes related to “positive regulation of the bile acid biosynthetic process”, “regulation of the lipid metabolic process”, and “the cholesterol metabolic process”, “cellular response to cholesterol”, “cholesterol homeostasis” indicating that the intake of quinoa diet (100 g/day) could influence lipid metabolism (Fig. 5B). Intriguingly, the expression profile of the HF + HQ group was “more abundant” than the expression profile of the HF + LQ group when compared with the expression profile of the HF group. The regulated genes were related mainly to “positive regulation of the triglyceride catabolic process”, “cellular response to interleukin-1”, “inflammatory response”, “cellular response to tumor necrosis factor”, “defense response to bacterium”, “positive regulation of lipoprotein lipase activity”, “phospholipid efflux”, “positive regulation of fatty acid biosynthetic process”, “cellular response to interferon-gamma”, “negative regulation of toll-like receptor 4 signaling pathway” and “response to oxidative stress”, indicating that increased intake of quinoa more effectively regulated the genes related to lipid metabolism, immune response, and oxidative stress (Fig. 5B).

We further verified the mRNA expression levels of some genes using qRT-PCR. The intake of quinoa (especially high amount in present study, equivalent to 300 g of daily human intake) more effectively increased the mRNA expression levels of some apolipoproteins, including Apoc2, Apoa4 (*p* < 0.01) and Apoa5 (*p* < 0.05), genes related to lipid metabolism. In addition, a high amount of quinoa effectively decreased the expression levels of Irf (interferon regulatory factor)5 and Tlr (Toll-like receptor)6, 10, 11, and 12 (*p* < 0.01) (Fig. 5C).

## Discussion

Quinoa is a pseudograin with a high nutritional value because it is rich in proteins, lipids, fibers, vitamins, and minerals and has an extraordinary balance of essential amino acids. In addition, quinoa possesses numerous secondary metabolites, such as saponins, phenolic acids, flavonoids, terpenoids, steroids, etc. These metabolites exhibit features beneficial to humans, including antidiabetic [16], anticancer [17, 18], immunoregulatory [19], and adjuvant activities [20].

In the present study, the rats were administered a HF diet and different amounts of quinoa (equivalent to 100 g/day and 300 g/day of human intake, respectively) simultaneously. The results showed that low amounts of quinoa, which is equivalent to 100 g/d of human intake, effectively controlled the weight of rats fed an HF diet. Several studies have investigated the effect of quinoa on weight. The vast majority of studies showed a positive association between quinoa consumption and weight decrease among animals [21], which may be beneficial to prevent hepatic steatosis induced by overweight. In fact, we found that quinoa reduced the TG and TC contents in liver tissues and mitigated pathological injury. However, more evidence and molecular mechanism investigations are still needed considering the multipathological factors in the progression of fatty liver.

A previous study showed that the consumption of quinoa was associated with decreased levels of serum cholesterol, TGs, low-density lipoprotein (LDL), and high-density lipoprotein (HDL) in rats fed a high fructose diet [22]. Lin et al. [8] investigated the protective effects of quinoa bran extracts on CCl_4_-induced liver injury and fibrosis in mice and found that red quinoa powder provided better protection than rutin against CCl_4_-induced oxidative stress, proinflammatory factor expression, and fibrosis development. Noratto et al. [15] found that quinoa intake reduced plasma and liver cholesterol, decreased obesity-associated inflammation, and prevented hepatic steatosis in obese db/db mice, which partially exhibited consistency with our results. The above results implied that quinoa may exert a positive role in the prevention of fatty liver.

Besides, oxidative stress changes play a very important role in the pathogenesis and progression of NAFLD. Due to insulin resistance and excessive mobilization of peripheral fat in NAFLD patients, a large amount of free fatty acids can be produced, leading to β-oxidation overload and an imbalance of the antioxidative system. This imbalance leads to lipid peroxidation, which can produce a large number of active and highly toxic intermediates, eventually affecting the normal function of liver cells [23]. In the present study, we found that quinoa effectively alleviated oxidative stress by increasing the activity of SOD and GSH-Px and decreasing the level of MDA under an HF diet. The antioxidant activity of quinoa has previously been investigated using several in vitro assays (such as the 2,2-diphenyl-1-picrylhydrazyl [DPPH] assay and ferric reducing antioxidant power [FRAP] assay) [24–26]; in addition, an *in-vivo* study showed that quinoa intervention could reduce the level of plasma lipid peroxidation and elevate the expression of antioxidant enzymes such as GSH-Px and CAT in several organs [27]. The antioxidative activity of quinoa could be attributed to its abundant phytochemicals, such as saponins, phenolic acids, flavonoids, terpenoids, and steroids [28]. Among these phytochemicals, saponins are one of the major phytochemicals present abundantly in quinoa seeds and contribute to the antioxidative activity of quinoa [29].

Moreover, fatty liver has been associated with unfavorable metabolic changes in circulation [30]. To further elucidate the beneficial effects of quinoa on lipid metabolism regulation under an HF diet model, we investigated the serum and hepatic metabolic profiles of rats fed an HF diet. Quinoa intake increased the levels of partial LysoPC (20:0, 20:1, 20:2, 22:5) and SM (d 18:0/24:1, d18:1/22:0) and decreased the levels of PC (18:0/18:1, 18:0/P-16:0, 16:0/18:1, 18:2/16:0).

LysoPC is an important component in human body and participates in many physiological functions. Barber et al. [31] revealed a generalized decrease in circulating LysoPC species in states of obesity, and their study suggested that diet and adiposity, rather than insulin resistance or diabetes per se, play an important role in altering the plasma Lyso profile. Another study suggested that circulating concentrations of different LysoPC species have been found to be reduced in diabetes and impaired glucose tolerance; for example, an increased level of LysoPC(20:2) was associated with a lower risk of type 2 diabetes [32]. In addition, a marked increase in plasma levels of PC has been reported in obesity [33]. Studies have found that serum LysoPC level in patients with cirrhosis and liver failure is also significantly reduced [34, 35]. A recent clinical metabolomic/lipidomic-based analysis of plasma showed that levels of PC were positively correlated and those of LysoPC were negatively correlated with hepatocellular ballooning in patients with NAFLD [36]. Therefore, the metabolic profile of LysoPC and PC in the quinoa intake group may reflect the improvement effects on hepatic steatosis from the aspect of lipid metabolism. However, the specific role of these lipid metabolites on gene expression and cell function still needs to be further investigated given the other conflicting results [37].

Furthermore, we also found that high amounts of quinoa (HF + HQ) increased the levels of serum γ-linolenic acid, linoleic acid, vitamin A, and hepatic pantothenic acid. A study demonstrated that γ-linolenic acid could be a potential marker to predict the future development of metabolic syndrome (MS) in obese subjects [38]. Another study showed that α-linolenic acid-enriched perilla oil suppressed HF diet-induced hepatic steatosis by ameliorating ER stress-mediated autophagy [39]. In addition, NAFLD may cause disturbed vitamin A metabolism [40], and the role of vitamin A in the occurrence and progression of fatty liver is contradictory. Oxidative stress plays a role in NAFLD pathogenesis and may increase consumption of vitamin A for antioxidant purposes; while a significant association was found between liver retinol concentrations and the histological classification of the disease [41].

To further investigate the molecular mechanism of quinoa on metabolism, we explored the mRNA expression profile of hepatic tissue. Low quinoa intake could regulate genes related to lipid metabolism, while high quinoa intake regulated mainly genes related to lipid metabolism and the immune response. Specifically, quinoa intake significantly upregulated the mRNA expression levels of apolipoproteins including Apoa4, Apoa5 and Apoc2, which are known to augment lipoprotein lipase (LPL) activity [42]. LPL is bound to the vascular endothelium and hydrolyzes chylomicron and VLDL-associated TG to facilitate the transport of hydrolyzed fatty acids to peripheral cells. Patients with genetic defects in Apoc2, Apoa5 or LPL display high circulating TG levels due to impaired TG clearance [42–44]. Moreover, quinoa intake significantly downregulated the mRNA expression levels of Irf5 and Tlr6, 10, 11, and 12. A recent study showed that elevated adipose Irf5 expression in obesity concurs with typical local and systemic inflammatory signatures. The upregulation of Irf5 may represent a novel adipose tissue marker for metabolic inflammation [45]. The gut microbiota is a source of Toll-like receptor (TLR) ligands, which can stimulate liver cells to produce proinflammatory cytokines under certain conditions. However, the liver has a high tolerance for TLR ligands because hepatic cells express minimal TLRs in normal liver. In contrast, TLR signaling is activated, and downstream molecules are increased in NAFLD because tolerance is disrupted [46]. Indeed, hepatic TLR expression is increased in human NAFLD patients, and in animal models, Tlr2, 4, 5, and Tlr9 have been shown to be associated with the pathogenesis of NAFLD [47–51]. Thus, TLRs are potential targets for NAFLD treatment. The above results primarily unveiled the molecular mechanism of the benefits of quinoa on NAFLD.

## Conclusions

In summary, our results demonstrated the beneficial effects of quinoa on weight gain control, improving hepatic steatosis and oxidative stress state under the HF diet style. These beneficial effects could be attributed to the regulation of the production of certain metabolites in the circulation system and the liver, including linoleic acid, LysoPC and PC, etc. In addition, quinoa intake effectively enriched the gene sets involved in lipid metabolism (such as apolipoprotein) and the immune response (such as toll-like receptors). However, more exploration is still needed on the pathway by which these genes are involved in the prevention effect of quinoa on NAFLD.

## Supplementary Information

**Additional file 1.** **Supplementary Table 1.** Nutrients composition of feed for SD rats in each group. **Supplementary Table S2.** Primer sequences for validation of genes.

## Data Availability

The data and materials that support the findings of this study are available from the corresponding author upon reasonable request.

## Abbreviations

NAFLD: Nonalcoholic fatty liver disease
NASH: Nonalcoholic steatohepatitis
LysoPC: Lysophosphatidylcholine
PC: Phosphatidylcholine
PE: Phosphatidylethanolamine
SM: Serum sphingomyelin
Apoa: Apolipoprotein
lrf5: Interferon regulatory factor
Tlr: Toll-like receptor
TG: Triglycerides
TC: Total cholesterol
SOD: Superoxide dismutase
GSH-PX: Glutathione peroxidase
MDA: Malondialdehyde
GSH: Glutathione
UPLC/Q-TOF–MS: Ultraperformance liquid chromatography-quadrupole time-of-flight mass spectrometry
