# Supplementary material for: Alleviating the effect of quinoa and the underlying mechanism on hepatic steatosis in high-fat diet-fed rats
Source: Nutr Metab (Lond). 2021 Dec 18;18:106. doi: 10.1186/s12986-021-00631-7 (PMC8684231; doi:10.1186/s12986-021-00631-7)
Supplement: Supplementary file 1 — Additional file 1. Supplementary Table 1. Nutrients composition of feed for SD rats in each group. Supplementary Table S2. Primer sequences for validation of genes. [file 12986_2021_631_MOESM1_ESM.docx]

**Supplementary Table 1**

**Nutrient composition of feed for SD rats in each group**

| Item | General diet | HF  (No quinoa) | HF+LQ  (9% quinoa) | HF+HQ  (27% quinoa) |
| --- | --- | --- | --- | --- |
| Energy (kJ/100 g) | 1481 | 2007 | 1970 | 1926 |
| Ash (g/100 g) | 5.0 | 3.1 | 3.6 | 2.9 |
| Protein (g/100 g) | 18.8 | 18.2 | 16.5 | 15.0 |
| Crude fiber (g/100 g) | 3.0 | 1.9 | 1.6 | 1.9 |
| Moisture (g/100 g) | 10.8 | 8.1 | 9.4 | 9.2 |
| Fat (g/100 g) | 5.7 | 26.3 | 25.8 | 23.2 |
| Carbohydrate (g/100 g) | 56.7 | 42.5 | 43.1 | 47.8 |

**Supplementary Table S2**

**Table S2 Primer sequences for validation of genes**

| GeneSymbol | Primers | 5’-3’ |
| --- | --- | --- |
| Apoa4 | Forward | CCAATGTGGTGTGGGATTACTT |
|  | Reverse | AGTGACATCCGTCTTCTGAAAC |
| Apoa5 | Forward | TCCTCGCAGTGTTCGCAAG |
|  | Reverse | GAAGCTGCCTTTCAGGTTCTC |
| Apoc2 | Forward | ATGGGGTCTCGGTTCTTCCT |
|  | Reverse | GTCTTCTGGTACAGGTCTTTGG |
| Irf5 | Forward | GGTCAACGGGGAAAAGAAACT |
|  | Reverse | CATCCACCCCTTCAGTGTACT |
| Tlr6 | Forward | AGCCAAGACAGAAAACCCATC |
|  | Reverse | GGGGTCATGCTTCCGACTAT |
| Tlr10 | Forward | TTTCCAGAGCTGCCAGGAAG |
|  | Reverse | AAGTTGGGGGACAGCACAAA |
| Tlr11 | Forward | TCCCTGATTGCATCATAGCAGA |
|  | Reverse | GGGCCGAGGTACAGAATGG |
| Tlr12 | Forward | CCTGGTCTCCCGCTATTTCAC |
|  | Reverse | CCGAGGTACAACTTCCAAGGT |
